# Duplicate Genitals with Labor

**Published:** 1913-11

**Authors:** 


Duplicate Genitals With Labor. J. E. Gemmell and A. M. Paterson, *Jour. Obs. Gyn. Brit. Emp.*, No. 23, 1913, describe a case with single anus and rectum, double vulva, vagina and uterus, bladder and urethra, wide separation of pubic bones and absence of umbilicus. Pregnancy and labor occurred in each uterus.

